# Supplementary material for: Hyperbolic phonon-polaritons in boron nitride for near-field optical imaging and focusing
Source: Nat Commun. 2015 Jun 26;6:7507. doi: 10.1038/ncomms8507 (PMC4491815; doi:10.1038/ncomms8507)
Supplement: Supplementary Information — Supplementary Figures 1-5. [file ncomms8507-s1.pdf]

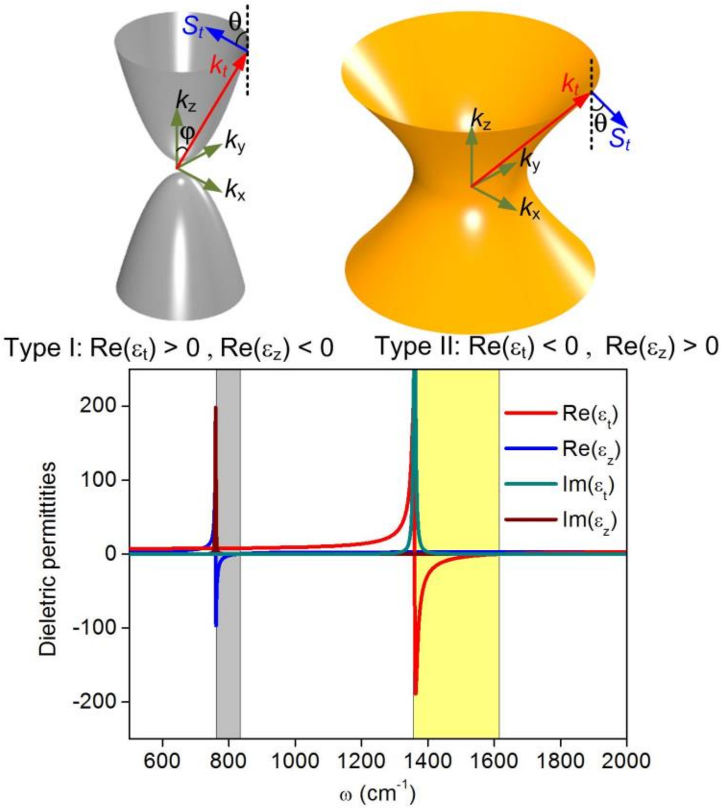

Supplementary Figure 1 | Hyperbolic dispersion in a hBN layer. Top, two types of hyperbolic dispersions (the Type I for Re(ε_t) < 0 and Re(ε_z) > 0 and the Type II for Re(ε_t) > 0 and Re(ε_z) < 0) and their corresponding vector diagrams. φ is the angle between the wavevector $\boldsymbol{k}_\mathbf{t}$ and z axis. θ is the angle between the Poynting vector $\boldsymbol{S}_\mathbf{t}$ and z axis. For large wavevectors ($k_x >> k_0$, $k_0 = 2\pi/\lambda$), $\varphi + \theta \approx \pi/2$. Bottom, in-plane and out-plane dielectric permittivities (ε_t , ε_z) of hBN. Two hyperbolic regions are marked in gray and yellow, respectively.

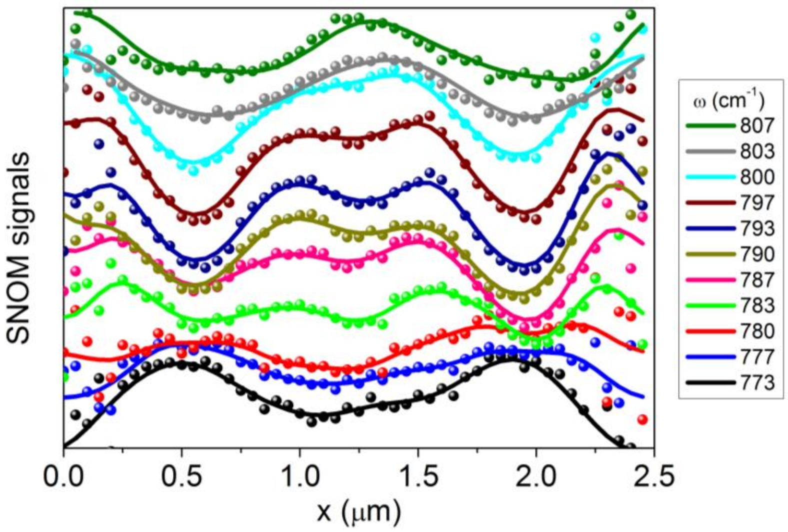

Supplementary Figure 2 | Experimental data of nano-FTIR line profiles with (solid lines) and without (color dots) the numerical smoothing.

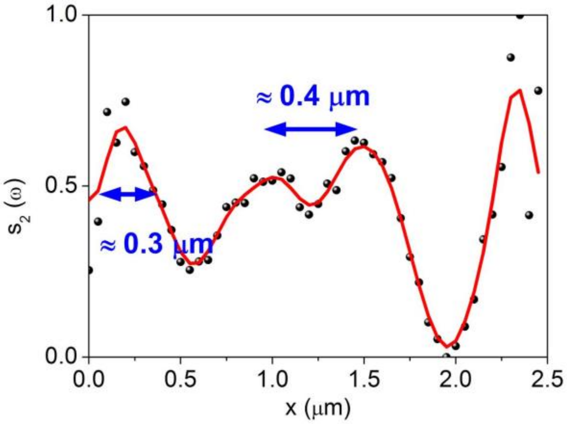

Supplementary Figure 3 | Experimental data of nano-FTIR line profiles with (solid lines) and without (black dots) the numerical smoothing at ω = 783 cm^{-1} (about 12. 8 μm). As mentioned in the main text, the smallest peak width of launched HPs that we can resolve in the Nano-FTIR line scans is about 0.3 μm at ω = 783 cm^{-1} (see the short, blue arrow), corresponding to a deep subwavelength scale of λ/42. However, for defining the optical spatial resolution, we consider the smallest resolvable peak-to-peak separation of about 0.4 μm (namely, λ/32 resolution).

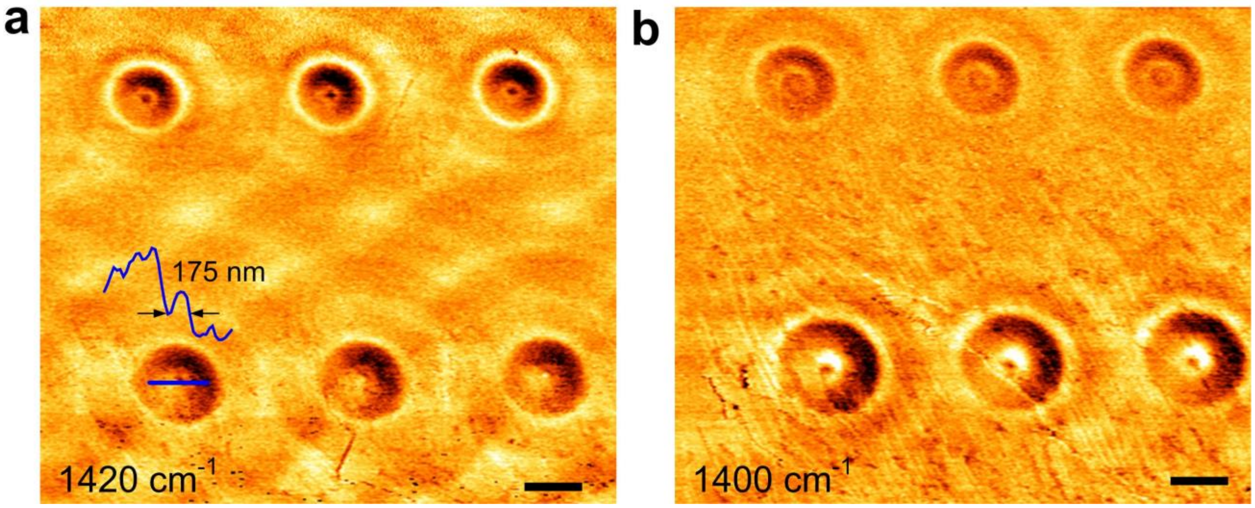

Supplementary Figure 4 | Experimental demonstration of near-field optical focusing and waveguiding of HPs. Near-field images of the nanodiscs (top row with diameter 500 nm, bottom row with diameter 750 nm) **a,** at 1420 cm^{-1} **b,** at 1400 cm^{-1}. Clear focusing and interference of HPs are found. The scale bars indicate 1000 nm. From the line profile across the focusing spot (shown in **a**, along the blue line), a width of about 175 nm (~ $\lambda/40$) of the spot are obtained.

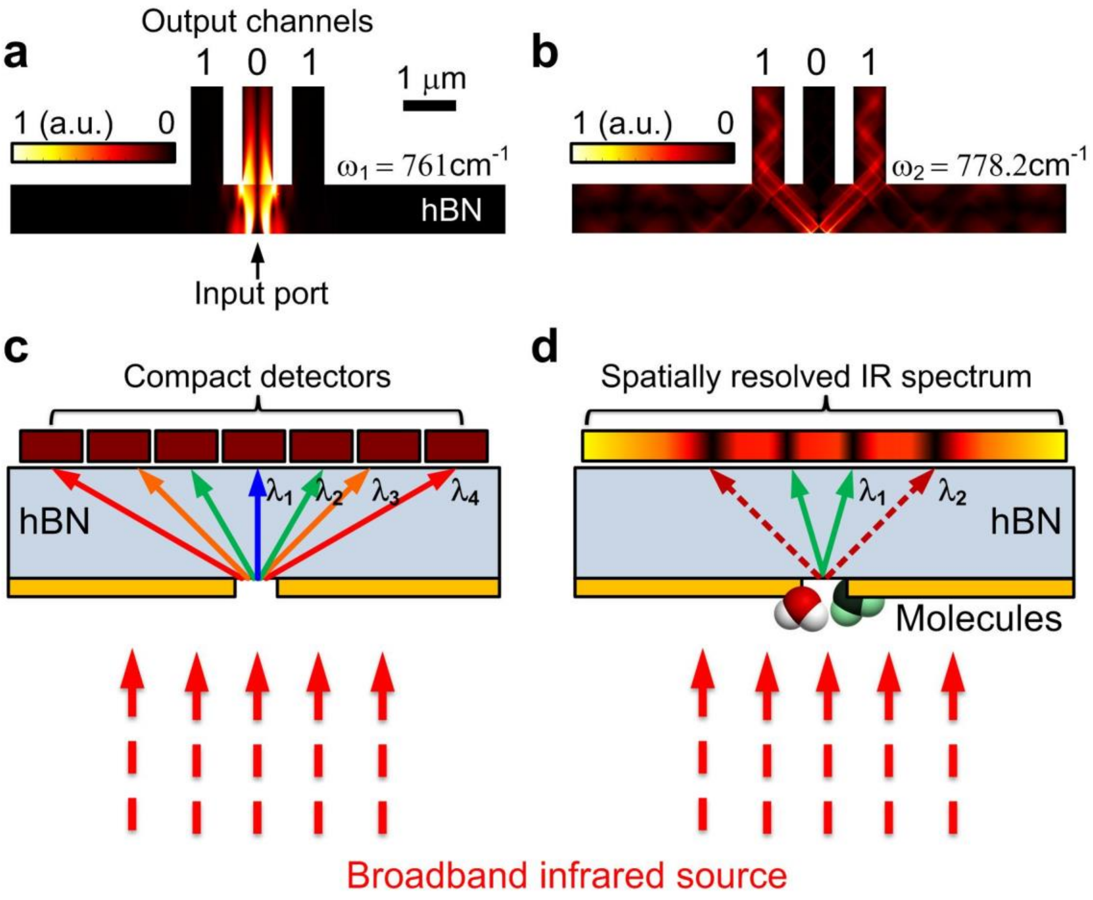

Supplementary Figure 5 | Further application proposals using the tunable highly directional HPs. Simulated $|E_z|$-distributions inside an hBN waveguiding structure (**a**) at $\omega = 761$ cm^{-1}, (**b**) at $\omega = 778.2$ cm^{-1}. Due to the small propagation angle, the electromagnetic fields primarily propagate into the inner '0'-channel. When tuning the frequency to $\omega = 778.2$ cm^{-1}, the fields propagate with an angle of ~45° and therefore transmit into the two outer '1'-channels. More complicated geometries could also expand such approaches beyond a binary routing into more sophisticated multi-channel systems. Consequently, this frequency-selective waveguiding could be useful for photonic switching or computing, infrared filtering, or various other nanophotonic applications. **c**, Sketches of an ultra-compact hBN-based infrared spectrometer. The thin hBN layer allows spatially splitting different wavelength components of the incoming infrared illumination. **d**, Sketches of hBN-based spatially-resolved infrared spectroscopy for different molecules. The dark colors in the spectrum represent the absorption by the molecules (sketch).
